# Fasting-mimicking diets as a strategy to reprogram tumor metabolism: a systematic review

**DOI:** 10.1007/s00394-026-03891-2

**Published:** 2026-02-12

**Authors:** Irislene Costa Pereira, Jorddam Almondes Martins, Dallyla Jennifer Morais de Sousa, Maria Shelda de Oliveira Neres, Rodrigo Soares Pereira Lima, Jheniffer da Silva Sousa, Rebeca Lima Monteiro, Felipe Cavalcanti Carneiro da Silva, Juliana Soares Severo, Francisco Leonardo Torres-Leal

**Affiliations:** 1 Metabolic Diseases, Exercise and Nutrition Group (DOMEN), Glauto Tuquarre Metabolic Diseases Laboratory (LabGT), Department of Biophysics and Physiology., Federal University of Piauí,, Teresina 64049-550, PI,, Brazil; 2 Laboratory of Genetical Toxicology, Center for Health Sciences, Federal University of Piauí, Teresina, Piauí Brazil

**Keywords:** Tumor metabolism, Fasting-mimicking diet, Stress resistance, Antitumor mechanisms

## Abstract

**Aims:**

The fasting-mimicking diet (FMD) is a plant-based, low-calorie dietary intervention characterized by reduced carbohydrate, with an emphasis on complex carbohydrates, and protein intake, alongside increased fat intake, designed to replicate the metabolic effects of fasting. Increasing evidence suggests that FMD may modulate tumorigenic pathways, enhancing the efficacy of anticancer treatments while minimizing adverse effects and resistance. This systematic review aimed to evaluate the effects of FMD on tumor metabolism, both as a standalone intervention and in combination with conventional and experimental therapies, and to identify the underlying mechanisms involved.

**Methods:**

This review was registered in PROSPERO and conducted according to PRISMA guidelines. A comprehensive search of Embase, PubMed/MEDLINE, Scopus, Web of Science, and Science Direct was performed using the term “fasting-mimicking diet.” Inclusion criteria comprised preclinical studies in mice or rats that implemented ≥ 50% caloric restriction and assessed the impact of FMD on tumorigenesis rate, tumor count, volume or weight, survival rate, inflammatory and immune responses, oxidative stress, gene/protein expression linked to antitumor effects, or treatment-related toxicity.

**Results:**

FMD alone was associated with delayed tumor progression, reduced metastasis, and downregulation of tumor-promoting biomarkers. When combined with chemotherapy, hormone therapy, targeted therapy, immunotherapy, or vitamin C, FMD enhanced antitumor efficacy through mechanisms involving oxidative stress modulation, improved antioxidant activity, autophagy regulation, and immune and inflammatory responses. These pathways contribute to the differential stress resistance observed in normal cells and increased vulnerability in tumor cells.

**Conclusion:**

Preclinical evidence suggests it can suppress tumor growth, reduce metastatic burden, and potentiate the therapeutic effects of multiple treatment modalities by modulating key metabolic pathways.

**Graphical abstract:**

**Supplementary Information:**

The online version contains supplementary material available at 10.1007/s00394-026-03891-2.

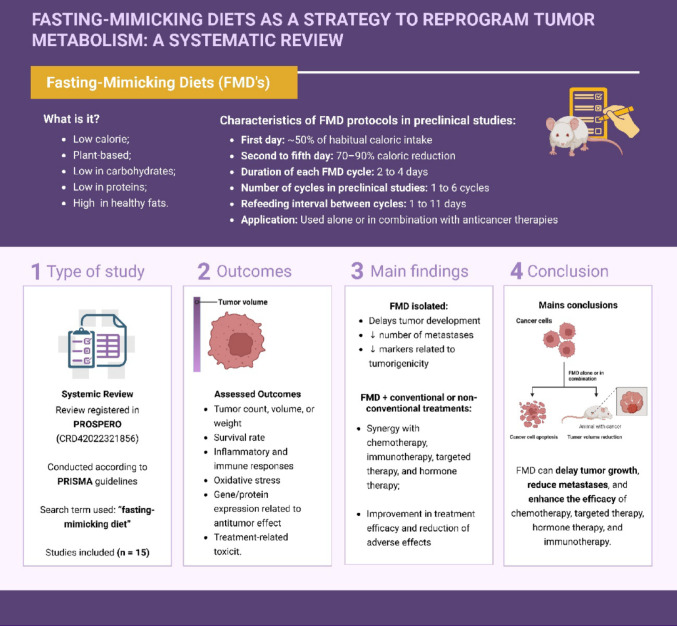

## Introduction

Cancer remains one of the leading causes of morbidity and mortality worldwide. Despite substantial advances in the understanding of tumor biology and the development of increasingly targeted therapies, the complexity and adaptability of cancer cells continue to pose significant clinical challenges [1, 2]. A hallmark of cancer is the remarkable ability of malignant cells to reprogram their metabolism, enabling sustained growth and survival even under conditions of metabolic stress [3, 4].

Metabolic reprogramming in cancer involves the activation of oncogenes such as *MYC*, *BRAF*, *RAS*, and *HIF-1*, as well as the loss of function in tumor suppressor genes including *TP53* and *PTEN*, thereby promoting a more aggressive phenotype [5, 6]. These alterations enhance energy production under unfavorable conditions such as hypoxia, elevated reactive oxygen species (ROS), and nutrient scarcity [4].

Understanding these metabolic alterations has led to the development of novel therapeutic strategies targeting metabolic pathways [7]. For instance, hyperactivation of the PI3K/AKT/mTOR pathway is associated with tumor progression and resistance to conventional therapies. However, pharmacological inhibitors of this pathway often cause severe side effects, such as hyperglycemia, which may lead to treatment discontinuation [8]. As a result, dietary interventions have emerged as complementary approaches to cancer therapy. Diet modulates systemic metabolic signals and provides nutrients that can support tumor growth [9], while influencing circulating levels of leptin, insulin, estradiol, and insulin-like growth factor 1 (IGF-1), all of which are implicated in tumor initiation and progression [10, 11].

Various nutritional strategies have been investigated in oncology, including calorie restriction, ketogenic diets, short-term starvation, and fasting mimicking diets (FMD) [12–17]. Among these, FMD has garnered increasing interest due to its ability to simulate fasting-induced metabolic states, thereby influencing cancer cell metabolism and therapeutic responsiveness [18–20]. Metabolic improvements, including reductions in body weight, abdominal and total body fat, and circulating IGF-1 levels, have been observed in individuals who followed a 5-day fasting-mimicking diet each month over a three-month period [21].

FMD is a plant-based dietary protocol characterized by short-term cycles (typically lasting 3 to 7 days) of caloric restriction, low protein and carbohydrate intake, and high fat content [22–25]. These cycles result in reduced glucose, IGF-1, and IGFBP-1 levels, alongside increased production of ketone bodies [21] thereby enhancing the antitumor effects of standard therapies. In this systematic review, we evaluated preclinical studies investigating the effects of FMD in experimental cancer models, with the aim of elucidating the underlying mechanisms by which FMD modulates tumor metabolism.

## Methods

### Data sources and search strategy

This systematic review was conducted in accordance with the PRISMA (Preferred Reporting Items for Systematic Reviews and Meta-Analyses) guidelines [26]. A preliminary search was first performed in PubMed/MEDLINE, Scopus, Web of Science, ScienceDirect, Embase, and PROSPERO to identify any published or ongoing systematic reviews on the topic. The review protocol was registered in PROSPERO under the identifier CRD42022321856.

A comprehensive search was then conducted in Embase, PubMed/MEDLINE, Scopus, Web of Science, and ScienceDirect, covering all records from inception to November 9, 2022, and updated on February 28, 2025. The search term used was “fasting mimicking diet,” without adding cancer-related terms to maximize retrieval and avoid missing relevant studies, following the strategy used by Ferguson et al. [27]. Additionally, the reference lists of retrieved studies and relevant review articles were manually screened for further eligible publications.

### Study selection

Four independent reviewers (ICP, JAM, DJMS, and MSON) screened titles and abstracts. Full-text articles of potentially eligible studies were then evaluated based on the inclusion and exclusion criteria described below. Discrepancies were resolved by consensus, and a fifth reviewer (FLTL) was consulted when needed.

### Inclusion criteria

Studies were included if they met all of the following criteria: (1) Preclinical studies using mouse or rat models; (2) Investigated the antitumor effects of FMD, either alone or in combination with other therapies; (3) Utilized an experimental cancer model; (4) Reported at least one of the following outcomes: tumor number, weight, or volume; animal survival rate; modulation of inflammatory markers; immune response; oxidative stress; expression of genes or proteins associated with antitumor effects; and/or adverse effects of conventional therapies, including impact on body weight.

### Exclusion criteria

Studies were excluded if they met any of the following criteria: Published in languages other than English or Spanish; Unpublished data; Clinical or in vitro studies; Did not assess the impact of FMD on antitumor parameters (Table 1).

**Table 1:** Description of the PICO criteria used in the present systematic review

Criteria	Description
Population	Animal Model of Cancer
Intervention	Fasting-mimicking diet alone or combination with other therapies
Control	Placebo or standard cancer therapy
Outcomes	Tumor number, weight, or volume; survival rate; modulation of inflammatory and immune responses; oxidative stress; expression of antitumor genes/proteins; adverse effects of treatment (including body weight)
Type of study	In vivo preclinical studies

### Risk of bias assessment

Each included study was independently evaluated by three reviewers (ICP, JAM, and MSON) using the SYRCLE risk of bias tool, an adaptation of the Cochrane Risk of Bias (RoB) tool specifically designed for animal intervention studies [28]. The tool assesses ten methodological domains across six categories of bias and classifies each study as having a “low risk of bias,” “some concerns,” or a “high risk of bias.

## Results

### General characteristics of eligible studies on FMD in experimental cancer models

**Fig. 1:**
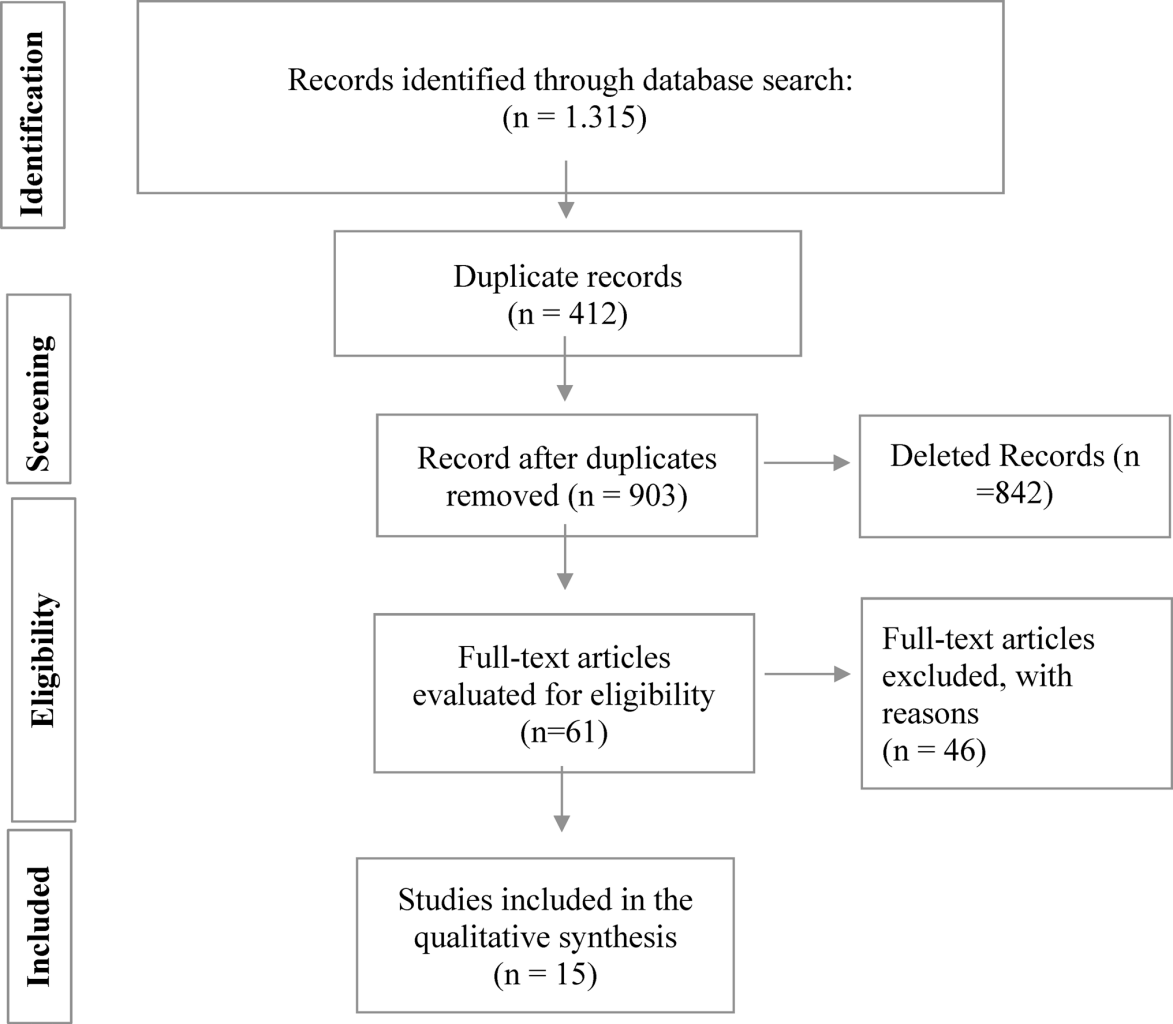
Flowchart of Search and Selection of Studies

A total of 1,315 records were initially identified. After removing 412 duplicates, 903 studies remained for title and abstract screening, of which 842 were excluded. Sixty-one full-text articles were assessed for eligibility, resulting in the inclusion of 15 preclinical studies in this systematic review, as illustrated in Figure 1.

**Fig. 2:**
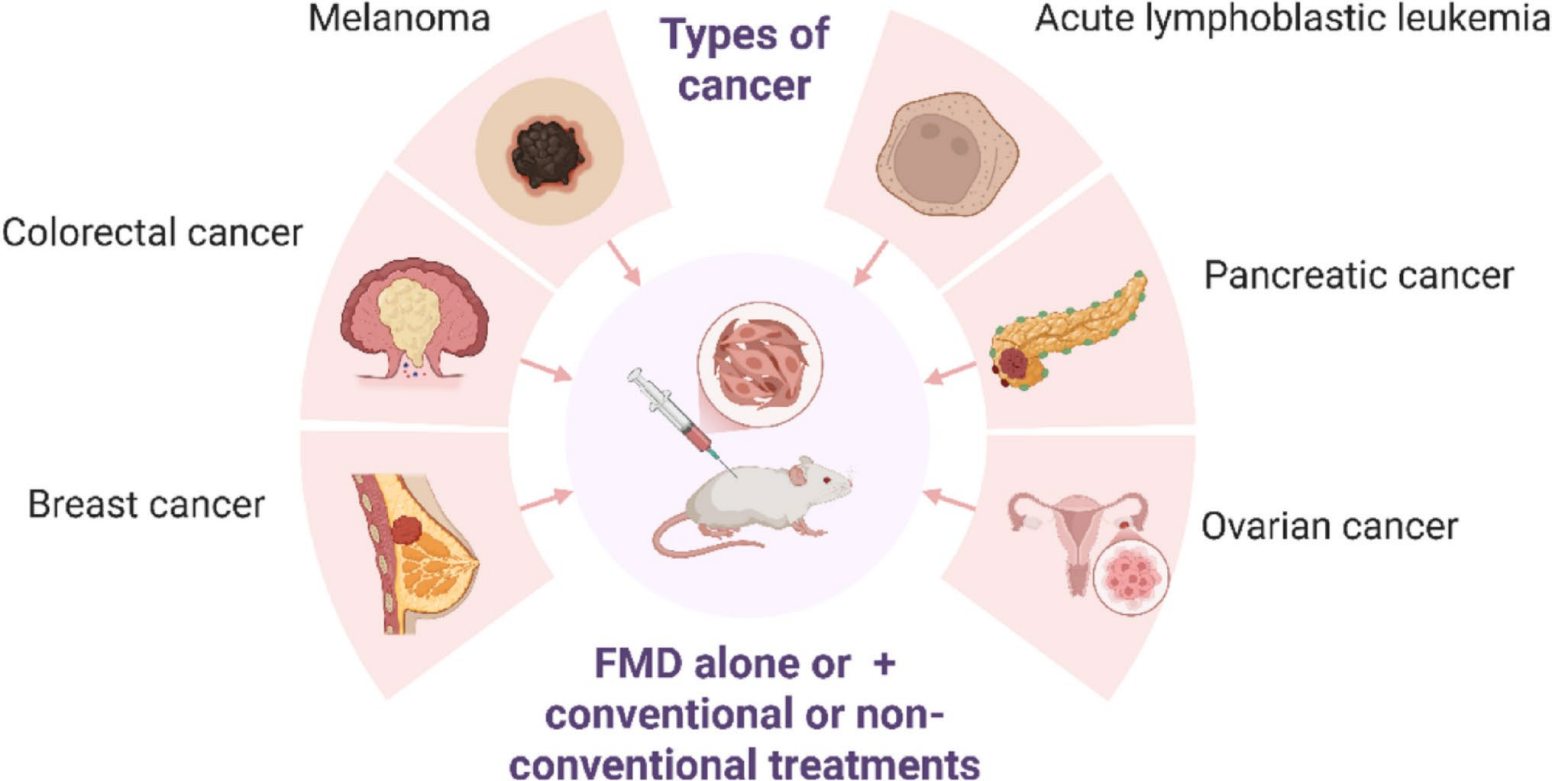
Experimental cancer models evaluated for the effects of the fasting-mimicking diet, alone or combined

All selected studies employed mouse models. Most investigations focused on breast cancer, particularly the triple-negative subtype. Additional models included acute lymphoblastic leukemia [29], ovarian cancer [30], colorectal cancer [12, 20, 31], melanoma [32] and pancreatic cancer [33] (Fig. 2). Tumor induction was achieved through injection of various cell lines, including BCR-ABL [34], 4T1, OVCA8, NCI/ADR-RES, MDA-MB-231, SUM159, MCF-7, HCT116, CT26, B16, and MC38. Notably, Li et al. [34] utilized cerulein to induce pancreatic carcinogenesis.

### Characteristics of the FMD protocols employed in the studies

Most studies implemented a standardized FMD protocol consisting of a 50% reduction in caloric intake on the first day, followed by a 90% reduction on days two to four [18, 31, 32]. However, notable variations existed. For instance, Pomatto-Watson et al. [35] applied a 70% caloric restriction after day one, while Zuo et al. [36] adopted a continuous 50-day FMD protocol. Li et al. [33] evaluated a regimen involving a 70% reduction over three days. The number of FMD cycles varied among studies. While some did not report the number of cycles [18, 37], others implemented 2 cycles [25, 30], 2–3 cycles [32, 38], 2–4 cycles [34, 35], or 4–5 cycles [39]. Cycle durations ranged from 2 to 4 days, with most studies employing 3- or 4-day protocols.

Refeeding durations also differed significantly. While Nan et al. [31], Li et al. [33], Buono et al. [34], and Wang et al. [38] applied 4 days of refeeding, Cortellino et al. [25] and Luo et al. [40] used 3 days. Pomatto-Watson et al. [35] implemented 10 days; Caffa et al. [18] used 5 and 7 days; and Di Biase et al. [32] prescribed 6–10 days (breast cancer) or 7–11 days (melanoma). Huang et al. [30] adopted a one-day refeeding period in both experimental protocols, while Raucci et al. [29] used six days (Tables 2, 3, 4).

**Table 2:** Characterization of eligible preclinical studies with animal cancer models subjected to FMD

References	Species (sex)	Type of cancer	Cancer induction method
Nan et al. [31]	Mice C57BL/6 (male)	Subcutaneous Colorectal Cancer	Injection into the distal posterior rectal submucosa with 1.0 × 10^6^ MC38 cells Intraperitoneal injection of azoxymethane (12 mg/kg), after one week, three cycles of sodium dextran sulfate (2.5% w/v) orally
Li et al. [33]	KC mice (LSL Kras G12D/+. Pdx1-Cre) (male)	Pancreatic cancer	Cerulein—50 μg/kg body weight, six injections in total with 1 h intervals between injections
Luo et al. [40]	Mice C57BL/6 (male)	Colorectal cancer	Subcutaneous Injection 2.0 × 10^6^ MC38 Cells
Raucci et al. [29]	Mice NOD SCID gama (NSG) (female)	Chronic lymphocytic leukemia	Intraperitoneal or subcutaneous injection of 1 × 10^11^ MEC1 cells
Buono et al. [34]	Mice C57BL/6 J	Acute lymphoblastic leukemia	Retro-orbital injection with 1.0 × 10^4^ BCR-ABL cells
Huang et al. [30]	Mice nu/nu (female)	Triple-negative breast cancer	Subcutaneous injection with 1.0 × 10^7^ MDA-MB-231 cells
		Ovarian cancer and resistant ovarian cancer	Subcutaneous injection with 1.0 × 10^7^ OVCA8 or NCI/ADR-RES
Wang et al. [38]	Mice BALB/c (female)	Triple-negative breast cancer	Subcutaneous injection of 5 × 10^5^/100 µL/mice of cells 4T1-Luc
Cortelino et al. [25]	Mice NSG and BALB/c (female)	Triple-negative breast cancer	Subcutaneous injection of 1.0 × 10^4^ cells 4T1 Subcutaneous injection of 1.0 × 10^5^ cells TS/A
Qian et al. [37]	Mice BALB/c (female)	Triple-negative breast cancer	Subcutaneous injection of 1.0 × 10^6^ cells 4T1
Pomatto-Watson et al. [35]	Mice BALB/cJ (female)	Triple-negative breast cancer	Subcutaneous injection of 1.0 × 10^5^ cell 4T1
Salvadori et al. [39]	Mice NOD/SCYD e Balb/c (female)	Triple-negative breast cancer	Subcutaneous injection of 2.0 × 10^4^ cells 4T1 Subcutaneous injection of 1.5 × 10^6^ cells SUM159
Caffa et al. [18]	Mice Balb/c nu—FoxN1 athymic and mice NOD/SCIDγ (female)	Hormone receptor positive breast cancer	Subcutaneous injection of 5.0 × 106 cells MCF-7 + 17β-estradiol releasing pellet in the intrascapular subcutaneous region Subcutaneous injection of 8.0 × 106 de cells ZR-75-1
Di Tano et al. [19]	Mice NSG and BALB/c	Colorectal cancer	Subcutaneous injection of 2.0 × 10 ^6^ of HCT116 in mice NSg and cells 2.5 × 10^4^ of CT26 in mices BALB/c
Di Biase et al. [32]	Mice BALB/c wild type and BALB/c- nu/nu e C57BL/6	Triple-negative breast cancer Melanoma	Subcutaneous injection of 2.0 × 10^6^ cells 4T1 Subcutaneous injection of 2.0 × 10^5^ cells B16

2-deoxy-D-glucose (2-DG), WZB117: 2-Fluoro-6-(m-hydroxybenzoyloxy) Phenyl m-Hydroxybenzoate; CSC: Stem cell escape; FMD: fasting-mimicking diet

**Table 3:** Characteristics of fasting-mimicking diet protocols in preclinical cancer studies

References	FMD Protocol	Cycles (days)	Cycle duration (days)	Refeed duration (days)	Associated therapy
Nan et al. [31]	50% day 1, 90% days 2–4	2	3	4	Yes
Li et al. [33]	70% for 3 days	3	3	4	No
Luo et al. [40]	50% day 1, 90% days 2–4	4	4	3	Yes
Raucci et al. [29]	50% day 1, 90% days 2–4	3	3	6	Yes
Buono et al. [34]	50% day 1, 10% days 2–3	4	3	4	Yes
Huang et al. [30]	50% day 1, 10% days 2–3	2	3	1	Yes
Wang et al. [38]	50% day 1, 10% days 2–3	3	3	4	Yes
Zuo et al. [36]	Continuous (50 days)	–			
Cortellino et al. [25]	50% day 1, 90% days 2–4	2	4	3	Yes
Qian et al. [37]	50% day 1, 90% days 2–4	4	4	3	Yes
Pomatto-Watson et al. [35]	50% day 1, 70% days 2–4	2 or 4	4	10	Yes
Salvadori et al. [39]	50% day 1, 90% days 2–4	4 or 5	4	*	Yes
Caffa et al. [18]	50% day 1, 90% days 2–4	2, 3, 4 or 6	2 and 4	5 or 7	Yes
		4	4	5 or 7	Yes
Di Tano et al. [19]	50% day 1, 90% days 2–4				Yes
Di Biase et al. [32]	50% day 1, 90% days 2–4	2 or 3	3 and 4	6 or 10	Yes
		2 or 3		7 or 11	Yes

FMD: fasting-mimicking diet

**Table 4:** Characteristics of therapies used in combination with a fasting-mimicking diet in preclinical cancer studies

References	Characteristics of the associated therapy				
	Classification	Substance	Dose	Frequency	Route of administration
Nan et al. [31]	Immunotherapy	anti-CTLA4	100 μg	Every 3 days	Intraperitoneal
Luo et al. [40]	Immunotherapy	Anti-PD1	100 μg	Every 3 days	Not specified
Buono et al. [34]	Chemotherapy	Vincristine	0.5 mg/kg	1×/ week	Intraperitoneal
	Antimalarial	Chloroquine	50 mg/kg	1×/ week	Intraperitoneal
Raucci et al. [29]	Proteasome inhibitor	Bortezomib	0.35 mg/kg	1×/week	Intraperitoneal
	Immunotherapy	Rituximab	10 mg/kg	1×/week	Intraperitoneal
Huang et al. [30]	Chemotherapy	Abraxane	10 mg/kg	2× with an interval of 4 days	Intravenous
		Doxil	10 mg/kg	2× with an interval of 4 days	Intravenous
Wang et al. [38]	Target therapy	Apatinibe	12.5 mg/kg	1×/day	Oral
		Liposomal clodronate	10 µL/g	1×/week	Intravenous
Zuo et al. [36]	Hormone therapy	Fulvestrant	100 mg/kg	2×/week for 4 week	Intramuscular
Cortellino et al. [25]	Immunotherapy	Anti-OX4	100 μg	3x/day on alternate days	Not specified
		Anti-PD-L	100 μg	3x/day on alternate days	Not specified
Qian et al. [37]		HCQ	15 mg/kg	4 doses with an interval of 4 days between each dose	Intravenous
Salvadori et al. [39]	Target therapy	WZB117	10 mg/kg	1×/day	Intraperitoneal
		2DG	500 mg/kg	1×/day	Intraperitoneal
		Metformin	150 mg/d	1×/day	Intraperitoneal
		Pictilisib	100 mg/kg	1×/day	Oral
		Palbociclib	62.5 mg/kg	Every two days	Oral
		Ipatasertib	75 mg/kg	1×/day	Oral
		Rapamycin	2 mg/kg	Every two days	Intraperitoneal
Caffa et al. [18]	Hormone therapy	Tamoxifen	45 mg	Daily	Oral
		Fulvestrant	150 mg	1×/week	Subcutaneous
		Palbociclib	65 mg/kg	3x/week	Oral
Di Tano et al. [19]	Vitamin and chemotherapy	Vitamin C	4 g/kg	2×/day	Intraperitoneal
		Oxaliplatin	10 mg/kg	1×/ every 15 days animals NSG 1×/a every 11 days animals BAlb/c	Intraperitoneal
Di Biase et al. [32]	Chemotherapy	Doxorrubicin	10 mg/kg	1× every cycle	Intraperitoneal
		Cyclophosphamide	150 mg/kg	1× every cycle	Intraperitoneal

**Fig. 3:**
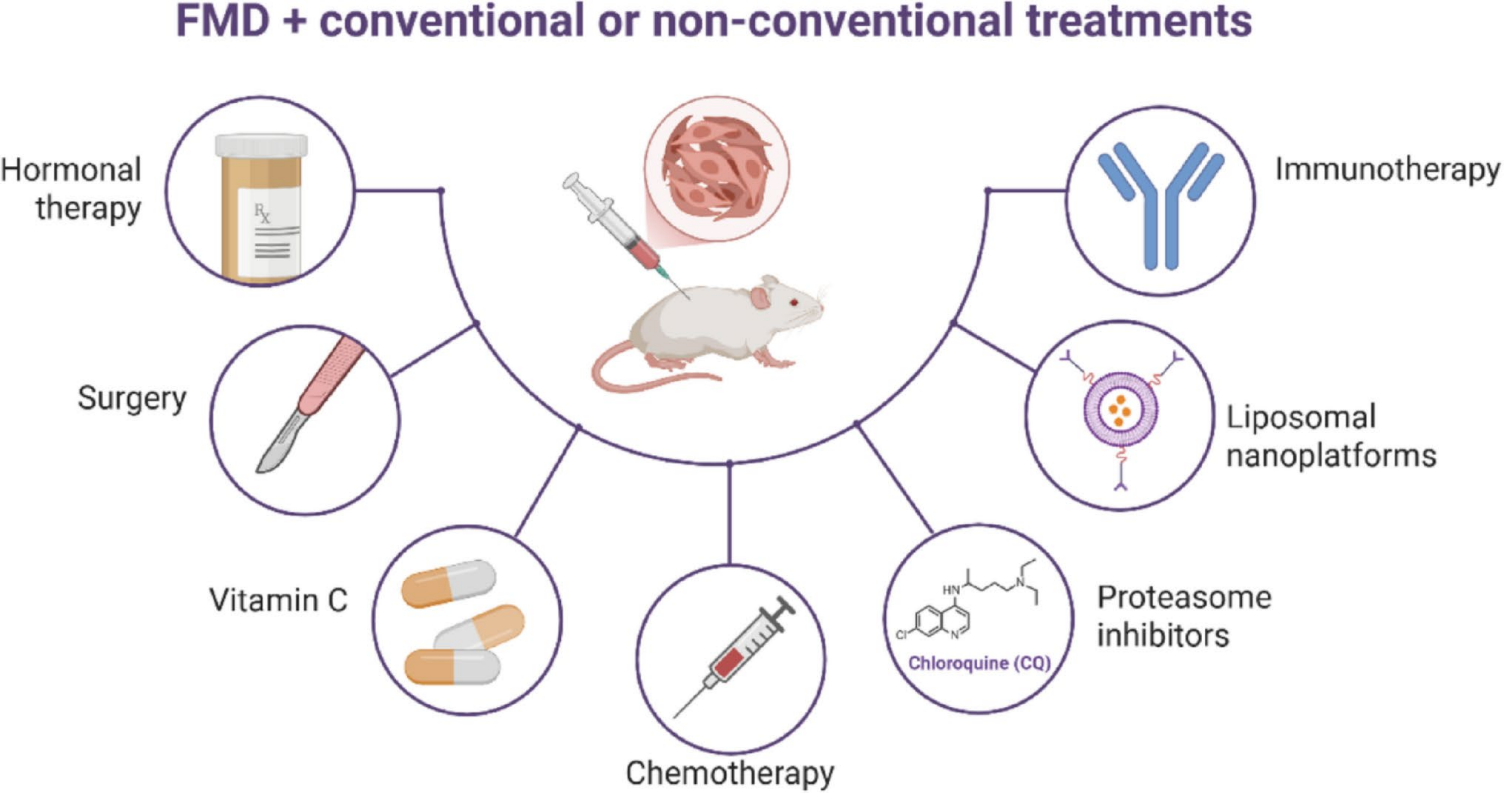
Types of treatments in which the fasting-mimicking diet (FMD) was combined for cancer therapy

FMD was combined with various therapeutic strategies, including immunotherapy [25, 29, 31, 40], hormone therapy [18, 36], targeted therapy [38, 39], chemotherapy [30, 32, 34], vitamin C [19], proteasome inhibitors [29], and liposomal nanoplatforms [34, 37] (Fig. 3). The purpose and mechanisms of each therapy are described in Supplementary Table 1.

### Risk of bias in studies investigating the effects of fasting-mimicking diet in animal cancer models

**Fig. 4:**
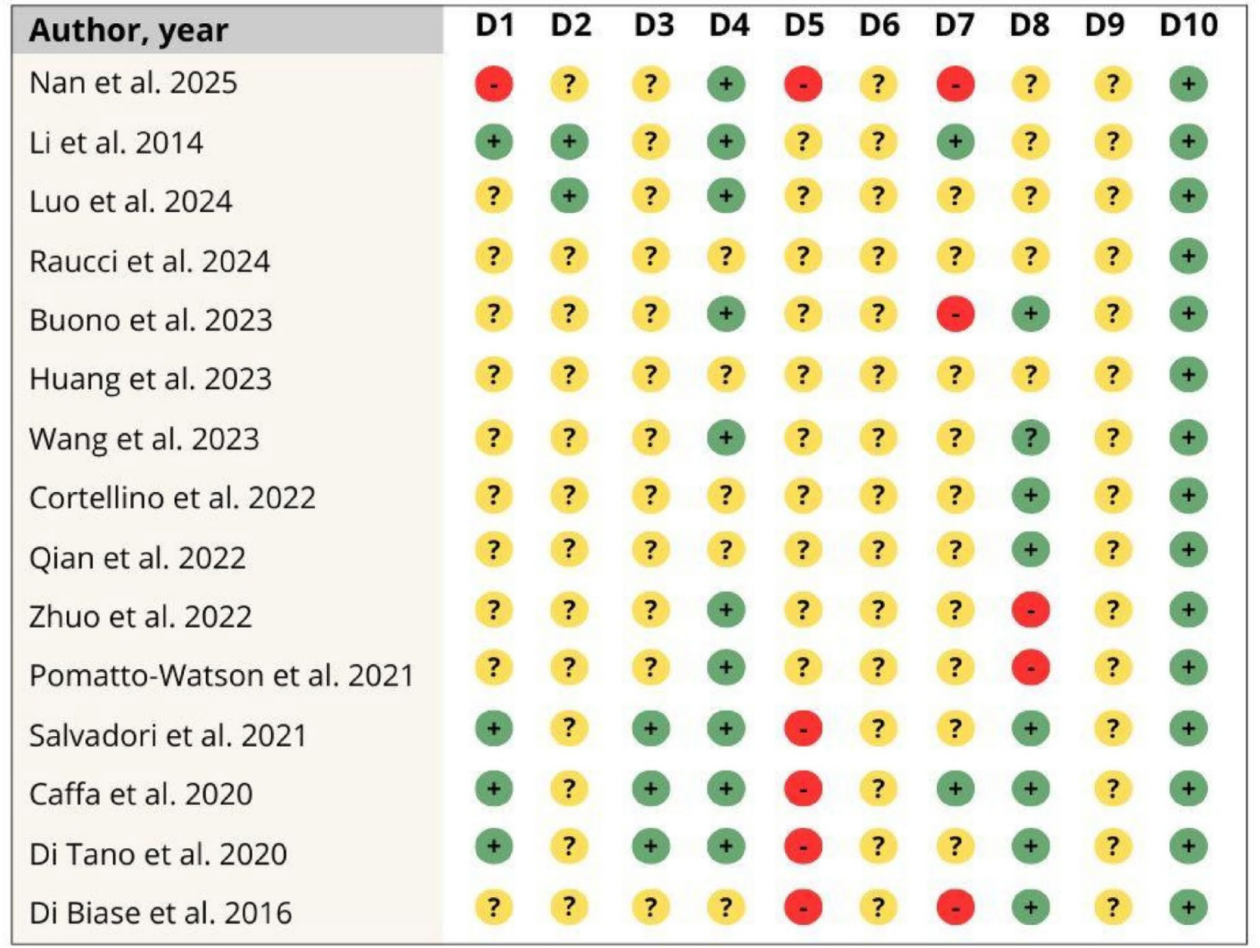
Risk of bias assessment of each study that investigated the effect of fasting mimicking diet in an animal model of cancer. D1: Sequence generation; D2: Baseline characteristics; D3: Allocation concealment; D4: Random housing; D5: Blinding; D6: Random outcome assessment; D7: Blinding; D8: Incomplete outcome data; D9: Selective outcome date; D10: Other sources of bias. Caption: + : low risk of bias; ?: some concerns; -: high risk of bias

**Fig. 5:**
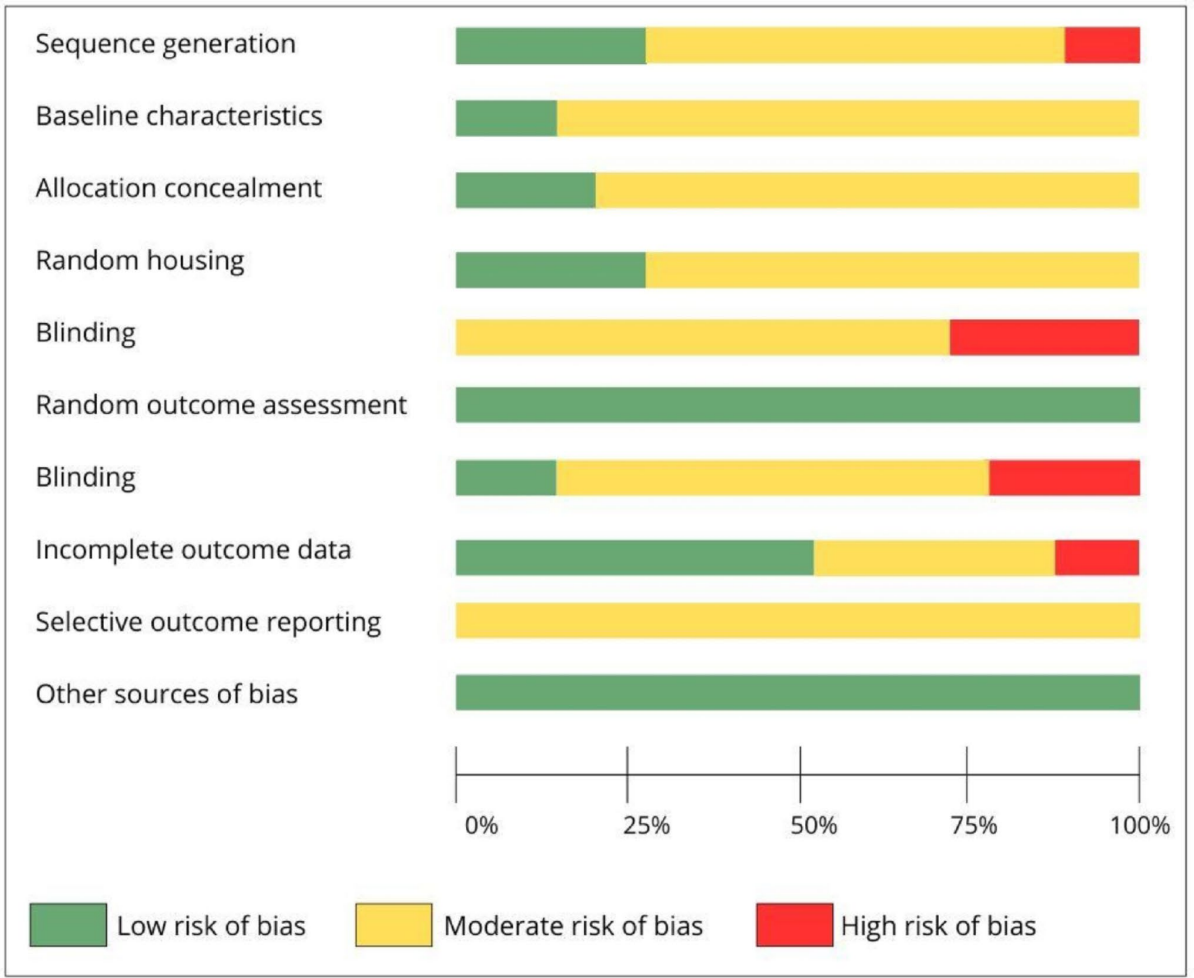
General risk of bias assessment of studies that investigated the effect of fasting mimicking diet in an animal model of cancer

Regarding individual risk of bias, domain 5—blinding—showed a high risk in approximately 33% of the included studies. Furthermore, most studies were classified as having an unclear risk of bias due to the lack of sufficient methodological details required to perform a robust assessment (Figs. 4 and 5).

### Effects of FMD alone or in combination with antitumor therapies

The effects of the fasting-mimicking diet (FMD) on cancer have been explored both in isolation and in combination with conventional and non-conventional therapies. Among the standard therapeutic strategies, FMD has been tested alongside chemotherapy, hormone therapy, immunotherapy, proteasome inhibitors, and targeted therapies. It has also been combined with emerging approaches, such as vitamin C and antimalarials. In all these contexts, studies have consistently demonstrated the potential of FMD to contribute to tumor control through diverse mechanisms.

When applied alone, FMD reduced tumor weight and volume in colorectal [19], breast [18, 32, 35, 39], and melanoma models [32]. In addition to delaying tumor progression and metastasis formation, FMD altered key regulatory molecules involved in proliferation: it downregulated pCREB, KLF5, and CCNB-CDK1, while upregulating CCND-CDK4/6. These findings support the role of FMD in inducing cell cycle arrest and disrupting survival pathways in tumor cells, thereby exerting a direct antiproliferative effect.

**Fig. 6:**
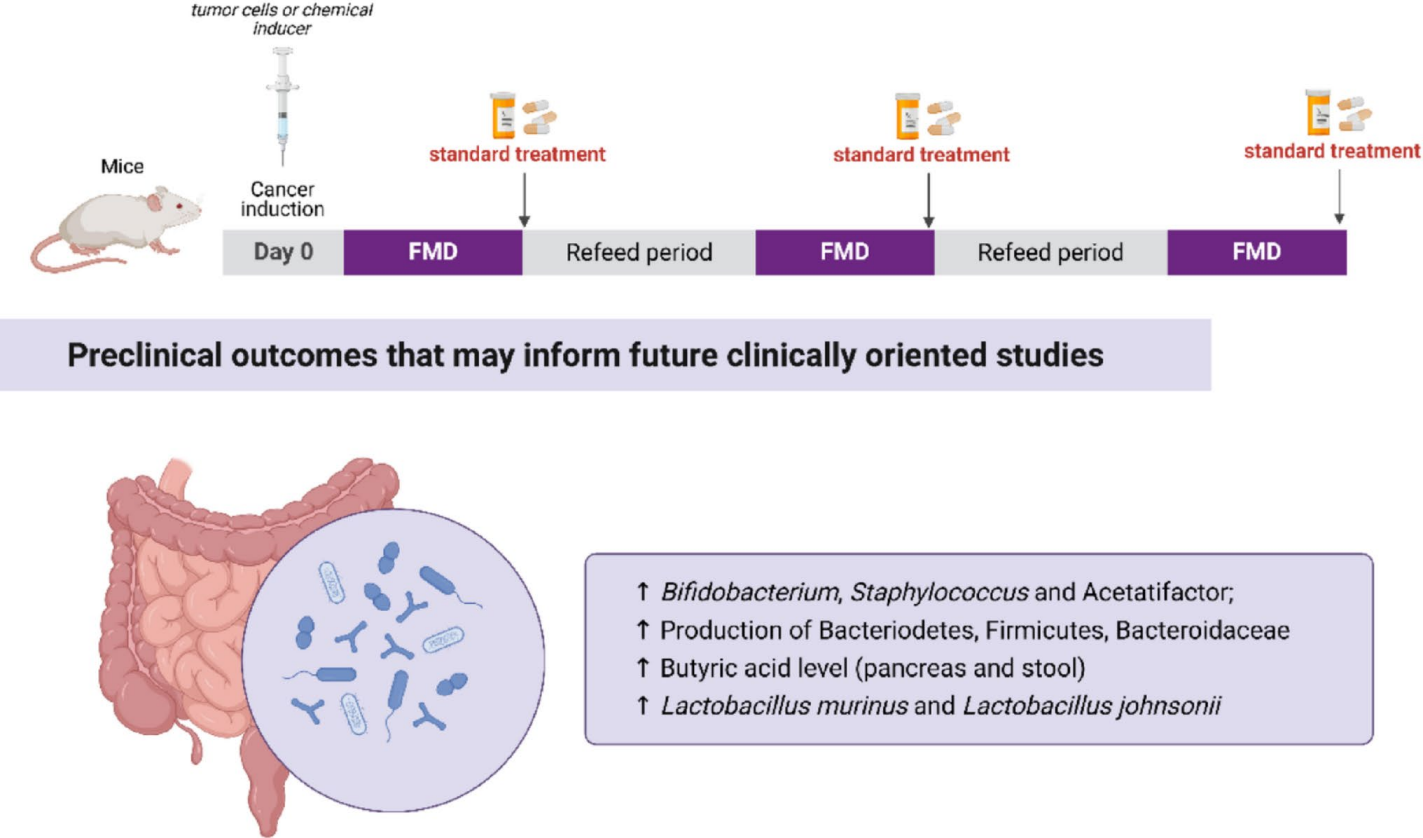
Effects of the included studies on FMD on the microbiota of cancer

In hormone receptor-positive breast cancer, FMD increased β-hydroxybutyrate, IGFBP1, IGF2R, and LC3-III levels, while reducing blood glucose, C-peptide, IGF-1, IGFBP3, leptin, TNF, and IL-1β [18, 37]. In models of colorectal and pancreatic cancer, FMD favorably modulated the gut microbiota, increasing the abundance of bacterial species associated with antitumor effects [31, 33, 40] (Fig. 6).

Furthermore, it enhanced immune responses, as evidenced by elevated CD8+ and CD25 + T cell populations [40]. These results suggest that FMD alone can modulate both metabolic and immunological pathways critical to cancer progression. Combining FMD with chemotherapy agents like doxorubicin and cyclophosphamide significantly enhanced tumor suppression in triple-negative breast cancer and melanoma models [32]. The synergy was reflected in increased tumor-infiltrating lymphocytes (CD3+/CD8+), elevated circulating CD8+ T cells, and a twofold rise in common lymphoid progenitors in bone marrow [32].

While ineffective alone in immunocompromised animals, FMD protected against chemotherapy toxicity in these models. It also reduced HO-1 expression in tumor tissue and increased it in non-tumor tissue, potentially limiting Treg function. In leukemia, the combination of FMD with vincristine improved survival regardless of obesity, regulated immune response, and reduced autophagy—an important mechanism of chemoresistance [34, 41].

The results from the leukemia study suggest that FMD enhances chemotherapy efficacy by reducing autophagy, boosting immune infiltration, and decreasing tumor burden while minimizing treatment-related toxicity [31]. Importantly, its effectiveness was maintained even in obese animals, indicating broad metabolic applicability. These benefits were consistently observed in models of breast cancer, leukemia, and melanoma, supporting the robustness of this dietary intervention across different tumor types and conditions.

FMD also potentiates the effects of hormone therapy. In breast cancer models, its combination with tamoxifen or fulvestrant increased PTEN expression, suppressed AKT phosphorylation, and downregulated mTOR, p70S6K, and eIF4E, while promoting 4E-BP1 activation [18]. This downregulation of the PI3K/AKT/mTOR pathway is significant given its role in tumor metabolism. FMD also reduced the expression of key regulators of cell cycle progression (E2F1, E2F2, CCNE1, CCND1), thereby promoting apoptosis and reducing tumor proliferation.

In addition, the FMD and palbociclib combination reduced fulvestrant resistance and tumor size. FMD also prevented tamoxifen-induced endometrial hyperplasia, as indicated by reduced uterine weight and histological abnormalities [18]. Similar effects were reported with fulvestrant in metastatic breast cancer, where FMD reduced hepatic glycogen storage and blood glucose [36]. These results underscore the potential of FMD to not only enhance hormone therapy efficacy but also mitigate associated side effects, reinforcing its relevance in endocrine-sensitive tumors.

When combined with PI3K/AKT and mTOR inhibitors, FMD enhanced therapeutic outcomes, preventing tumor progression for over 150 days in 85% of mice and delaying the onset of resistance. The combination also restored blood glucose homeostasis during treatment, a critical advantage given the hyperglycemia often associated with these inhibitors [39]. In a breast cancer model, FMD acted synergistically with apatinib (a tyrosine kinase inhibitor), reducing tumor-associated macrophage infiltration and overall cancer progression [38]. These data demonstrate FMD’s capacity to modulate both immune and metabolic responses, strengthening its role as a therapeutic adjuvant.

**Fig. 7:**
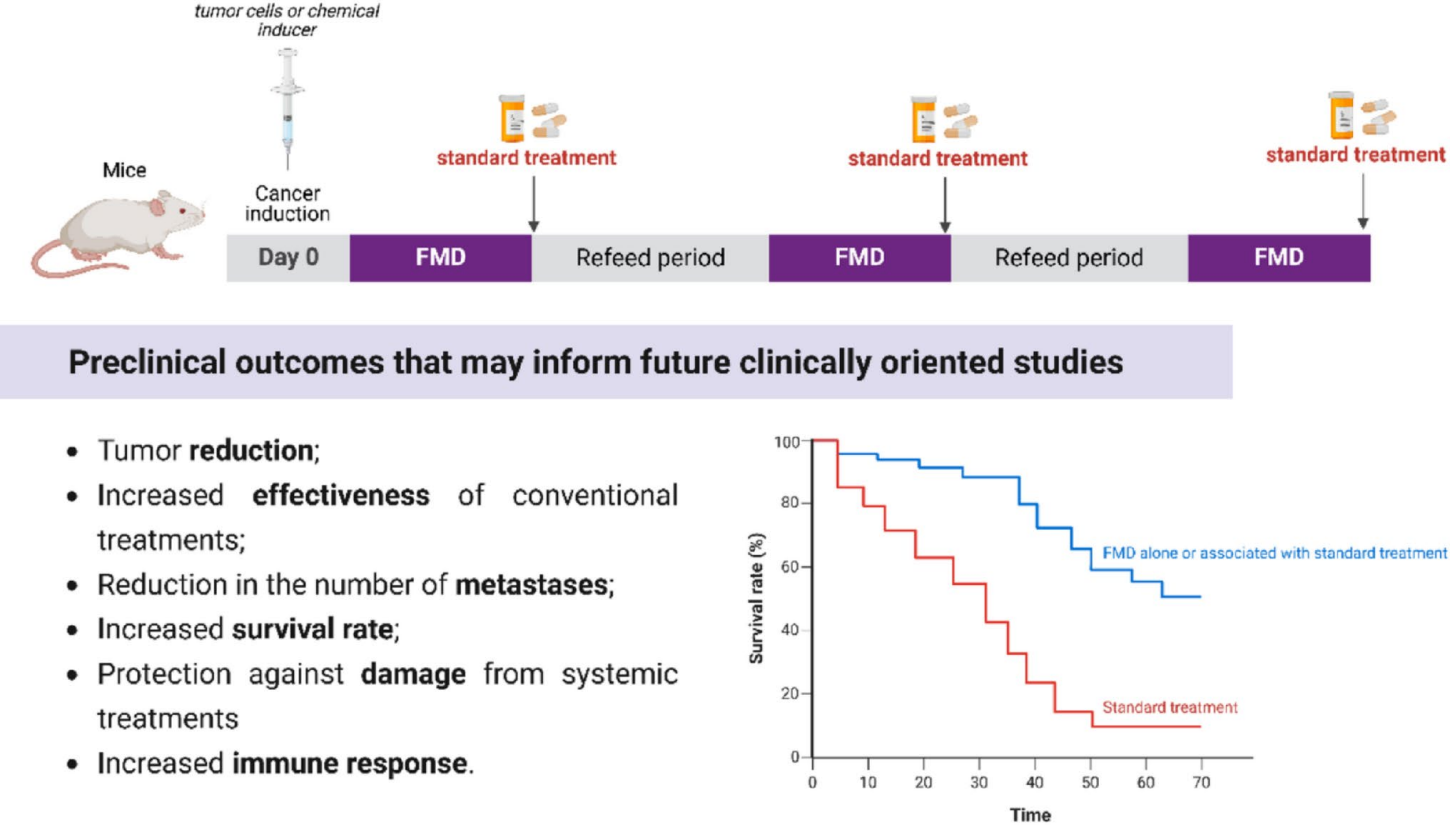
Effects of FMD on the modulation of outcomes that increase the survival of animals with cancer

In the context of immunotherapy, which has revolutionized cancer treatment through checkpoint inhibitors, CAR-T cells, and cancer vaccines [42], FMD has shown additional benefits. Its combination with immunotherapy delayed tumor progression and prolonged survival (Fig. 7) by approximately seven days. It also increased γδ T-cell infiltration, promoted their differentiation into cytotoxic phenotypes, and reduced splenomegaly [25] further highlighting its immunomodulatory effects. 

Additionally, FMD remodeled the tumor microenvironment, reducing stromal density and collagen deposition, normalizing tumor vasculature, and decreasing pericyte and endothelial cell populations. In low-immunogenic breast cancer models, FMD enhanced the response to immunotherapy, despite showing limited effects when used alone [25]. Although data are still limited, these findings support the hypothesis that FMD sensitizes tumors to immunotherapy by reshaping the immune landscape and tumor stroma.

FMD has also been explored in combination with emerging nonstandard therapies. When paired with high-dose vitamin C, it enhanced iron-dependent tumor toxicity by reducing FTH expression and increasing oxidative stress [19]. Similarly, its association with hydroxychloroquine, delivered via a nanoplatform, effectively reduced tumor growth and autophagosome formation, with no observed damage to critical organs such as the liver, kidney, lung, or heart [37]. These results suggest that FMD can safely potentiate the effects of experimental therapies.

Concerns surrounding the potential for malnutrition due to caloric restriction were not substantiated in the reviewed studies. Animal models subjected to FMD exhibited a temporary weight loss followed by a return to the normal weight upon resuming the standard diet. In the studies analyzed, the recovery period ranged from 1 to 11 days, with 3 days being sufficient for body-weight restoration without compromising the animals’ nutritional status (Table 5). Furthermore, FMD prevented specific adverse effects associated with standard therapies. It reduced splenomegaly and anaphylaxis risk during immunotherapy [25], prevented tamoxifen-induced endometrial hyperplasia [18] and normalized blood glucose during PI3K/AKT/mTOR inhibition [39].

**Table 5:** Effect of a fasting-mimicking diet on an experimental cancer model

References	Type of treatment (type of cancer)	Main outcomes
Nan et al. [31]	FMD isolated FMD + anti-CTLA-4 (Colorectal cancer)	*FMD isolated:* Weight loss did not exceed 20% during the FMD cycle ↓ Tumor weight and size and tumor burden ↑ Lactobacillus, Bifidobacterium, GCA_900066575, Staphylococcus e Acetatifactor ↑ CD8+ and CD25 T cells *FMD + anti-CTLA-4:* FMD improved the efficacy of anti-CTLA-4 therapy, further reducing tumor weight and tumor size, compared to anti-CTLA-4 therapy alone
Li et al. [33]	FMD isolated (Pancreatic cancer)	↓ disease progression; ↓pancreatic injury ↑ expression of ACOX1 ↑ Production of Bacteriodetes, Firmicutes, Bacteroidaceae ↑ Butyric acid level (pancreas and stool)
Luo et al. [40]	FMD isolated and FMD + Anti-PD-1 (Colorectal cancer)	*FMD isolated:* ↓ tumor volume, ↓ Tumor weight, ↓ Ki-67, ↓ CD31 ↑ % of CD45⁺ in the tumor, ↑ % of CD8⁺/CD3⁺ in the tumor and peripheral blood ↑ TNF-α, ↑ *Lactobacillus murinus* and *Lactobacillus johnsonii* *FMD + Anti-PD-1* ↓ tumor volume and tumor weight
Raucci et al. [29]	FMD + 26S proteasome inhibitors + immunotherapy (Leukaemia)	↓ Spleen weight ↓ number of leukemic lymphocytes in bone marrow, spleen, peripheral blood, and peritoneal exudate ↓ malignant lymphocytes in all organs and tissues analyzed, ↑ survival of animals
Buono et al. [34]	FMD + chemotherapy (Acute lymphoblastic leukemia)	↑ survival of normal weight and obese animals; ↓ reduced the number of GFP + tumor cells in spleens. FMD + vincristine contributed to anticancer immunity
Huang et al. [30]	FMD + chemotherapy (Breast cancer, ovarian cancer and resistant ovarian cancer)	Caused a large shrinkage or reduction of cell nuclei ↓ cell proliferation, regardless of the type of nanodrug used ↑ cell apoptosis, regardless of the type of nanodrug used
Wang et al. [38]	FMD + target therapy (Breast cancer)	↓ tumor volume, ↓ lung metastasis, ↓ M2 tumor-associated macrophage (TAM) infiltration, ↓ lactic acid production FMD promoted inhibition of mTOR phosphorylation and HIF-1α expression
Zuo et al. [36]	FMD + hormone therapy (Metastatic breast cancer)	↓ liver metastasis nodules, ↓ metastatic burden in mice with metastatic tumors FMD prevented the accumulation of glycogen in the liver and blocked the rise in glucose
Cortellino et al. [25]	FMD + immunotherapy (Triple negative breast cancer)	↓ tumor volume, ↑ longer survival, ↓ splenomegaly, Promoted activation and differentiation of T lymphocytes, ↓ Immunosuppressive PMN-MDSCs in the immune infiltrate, Remodels tumor stroma and normalizes tumor vasculature ↓ risk of anaphylaxis associated with immunotherapy, however, immunodeficient mice enjoyed this benefit ↑ efficacy of anti-tumor response induced by anti-OX40/anti-PD-L1, ↑ animal survival, modulation of CD4 T cell activity, ↓ immunosuppressive M2 macrophages,
Qian et al. [37]	FMD + targeted lysosomal delivery nanoplatform (Triple negative breast cancer)	↓ tumor growth, ↑ extensive necrosis in tumors, ↑ autophagosomes in tumor cells ↑ expression of LC3-II or SQSTM1, absence of changes in the liver, kidney, lung or heart
Pomatto-Watson et al. [35]	FMD isolated (Triple negative breast cancer)	↓ tumor growth rate, ↓ tumor area, ↓ tumor size, ↓ IGF-1 levels No significant loss of lean mass or fat mass was observed ↓ percentage of splenic FoxP3+ CD4+ Treg
Salvadori et al. [39]	FMD + target therapy (Triple negative breast cancer)	↓ percentage of CD44+ and CD24 cells, ALDH1, CSC There was a delay in the formation of metastasis and prevented it from occurring in several organs. ↓KLF5, CREBp, G9A, H3K9 2DG potentiated the effect of FMD in delaying tumor progression, ↓ mammospheres derived from tumor masses, ↑ survival, prevented the formation of tumors for more than 150 days The association with PI3k/Akt/mTOR inhibitors had better antitumor action, with ↑ progression-free survival, preventing tumor development for more than 150 days in 85% of animals, ↓ KLF5 and H3k9me2. Furthermore, it prevented the development of hyperglycemia and delayed the development of resistance ↓ mammosphere number, ALDH1, ↓ tumor volume, ↑ caspase 3, ↓ GLUT1, KLF5, CREBp, G9A, H3K9
Caffa et al. [18]	FMD + hormone therapy (Hormone receptor positive breast cancer)	↓ tumor volume, ↓ levels of IGF-1, C-peptide, leptin, IGFBP3, TNF, IL-1β, and pAkt, E2F1, E2F2, CCNE1, CCND1, RB ↑ IGFBP1, HMB, adiponectin, PTEN, 4E-BP1, EGR1 ↓speed of tumor growth, ↓ tamoxifen-induced endometrial hyperplasia, ↓ intra-abdominal fat
Di Tano et al. [19]	FMD + vitamin C (Colorectal cancer)	↓ tumor volume, ↓ HO-1 in the tumor, ↑ serum iron
Di Biase et al. [32]	FMD + chemotherapy (Triple negative breast cancer)	Slow tumor growth, ↓ IGF-1 Sensitization to doxorubicin and cyclophosphamide ↑ 33% in circulating CD8+ lymphocytes, ↑ animal survival, ↓ HO-1 in the tumor ↑ HO-1 serum, ↑ number of CD3+/CD8+ TILs
	FMD + chemotherapy (Melanoma)	Had an additive effect on tumor suppression, ↓ tumor volume, ↑ CD3+/CD8 TILs

ALDH1A1: Aldehyde Dehydrogenase 1 Family Member A1; CCND1: Cyclin D1; CCNE1: Cyclin E1; CLP: Common lymphoid progenitors; FMD: Fasting mimicking diet; CREBp: cAMP Response Element-Binding Protein; CSC: cancer stem cell; E2F1: E2F transcription factor 1; E2F2: E2F transcription factor 2; EGR-1: Early growth response protein 1; EIF4EBP1: Eukaryotic translation initiation factor 4E binding protein 1; G9A: histone methyltransferase G9a; H3K9: Histone H3 lysine 9; HCQ: hydroxychloroquine; HMB: Beta-hydroxy beta-methylbutyrate; HO-1: Heme oxygenase-1; IGF-1: Insulin-like growth factor 1; IGFBP-1: Insulin-like growth factor binding protein 1; IGFBP-3: Insulin-like growth factor binding protein 3; IL-1β: Interleukin-1B; KLF-5: KLF Transcription Factor 5; mTOR: protein of rapamycin in mammals; PD-L1: Myocardial programmed death-ligand 1; PMN-MDSCs: polymorphnuclear myeloid-derived suppressor cell; PTEN: Phosphatase and tensin homolog; RB: retinoblastoma protein; TILs: Tumor-infiltrating lymphocytes; TNF: Tumor Necrosis Factor Alpha

## Discussion

This systematic review comprehensively examined the effects of fasting-mimicking diets (FMD), both as monotherapy and in combination with antitumor agents, across different cancer models, including breast, colorectal, pancreatic, leukemia, ovarian, and melanoma. Consistently, FMD reduced tumor size, area, and volume, slowed disease progression, decreased metastasis incidence, and modulated metabolic and hormonal markers involved in tumor promotion, such as insulin, glucose, IGF-1, and leptin. These effects were further amplified when combined with chemotherapy, hormone therapy, immunotherapy, vitamin C, and targeted therapies.

One of the central mechanisms associated with these results involves the reduction of circulating IGF-1 levels, which plays a key role in metabolic regulation by controlling glucose uptake, lipid metabolism, and insulin homeostasis [43]. Elevated IGF-1 concentrations are strongly associated with the development and progression of several types of cancer [44–48], and in breast cancer, increased IGF-1 is linked to the activation of pro-tumoral pathways such as Ras/Raf/MAPK and PI3K/Akt. These pathways promote cell proliferation, tumor survival, and therapeutic resistance [49–51]. Thus, the metabolic modulation promoted by FMD contributes to reducing the activation of these pathways and mitigating angiogenesis, inflammatory cell recruitment, and treatment resistance [52, 53], establishing a first axis of tumor microenvironment remodeling.

However, it is important to recognize that FMD-induced inhibition of PI3K/Akt and mTOR signaling does not occur uniformly across all cell types. In some contexts, particularly in more differentiated cancer cells, compensatory activation of these pathways may occur as a mechanism of escape from nutrient deprivation, suggesting that the combined use of FMD with specific inhibitors of these pathways may be a viable approach [39]. Thus, although the predominant trend in preclinical models is inhibition of these axes, their compensatory activation in certain contexts reinforces the relevance of combined therapeutic strategies.

In parallel, nutrient restriction induced by FMD triggers conserved adaptive responses. Energy limitation inhibits Ras-PKA and Tor-S6K pathways, while increasing the expression of stress-resistance genes such as SOD2 and catalase [32]. This antioxidant and cytoprotective response plays an important role in protecting cells against damage, including chemotherapy-induced injury. In this same context of oxidative stress, modulation of heme oxygenase-1 (HO-1) by FMD becomes particularly relevant: although HO-1 exerts protective effects in normal cells, its overexpression in tumors is associated with progression, angiogenesis, metastasis, and therapeutic resistance [54–56]. Studies show that HO-1 inhibition sensitizes tumors to chemotherapy in different experimental models [57–59], and its integration into the set of adaptive responses modulated by FMD suggests an additional mechanism for reducing tumor aggressiveness.

These oxidative stress-related changes and the broader set of adaptive responses provide insight into the contrast between differential stress resistance (DSR) in normal cells and differential stress sensitization (DSS) in cancer cells. In normal cells, energy is redirected toward protective mechanisms, promoting tolerance to damage; in tumor cells, hyperactivation of oncoproteins such as Ras, PKA, and Tor prevents an effective adaptive response, making cancer cells more susceptible to growth inhibition and apoptosis [32]. This duality simultaneously explains the reduction in systemic toxicity and the increased antitumor efficacy observed in studies combining FMD with cytotoxic therapies.

This metabolic remodeling and increased tumor sensitivity to stress directly affect the immune system. FMD enhances the antitumor immune response by increasing circulating CD8+ lymphocytes, common lymphoid progenitors (CLPs) in the bone marrow, and CD3+/CD8+ tumor-infiltrating lymphocytes (TILs) [32]. Additionally, it reduces the proportion of FoxP3+ regulatory T cells, promoting a microenvironment more responsive to immunotherapy [35]. The enhancement of TIL infiltration and functionality is particularly important, as these lymphocytes constitute a key component of antitumor immunity. Although TIL-based therapies involve high cost and complexity [60], FMD emerges as a low-cost strategy to potentiate this pathway, increasing the effectiveness of immunotherapeutic approaches.

The intensification of the immune response, together with metabolic and oxidative-stress-related alterations, also converges to influence processes related to metastasis, responsible for roughly 90% of cancer-related deaths [61]. Although FMD does not completely eliminate metastasis formation, several studies demonstrate its ability to substantially reduce metastasis incidence, both alone and in combination with targeted therapies [18, 35]. In models using 4T1 cells, which are highly metastatic, FMD reduced metastatic foci by 80%, restricted to the lungs, whereas animals fed ad libitum displayed metastatic lesions in the liver, spleen, ovaries, and lymph nodes [18]. These findings highlight the influence of FMD on the tumor microenvironment and systemic immunity, critical factors in tumor dissemination [62], and raise the possibility that the diet may potentiate phenomena such as concomitant immunity, helping limit the establishment of secondary metastases, particularly in breast cancer subtypes with distinct metastatic patterns [63].

**Fig. 8:**
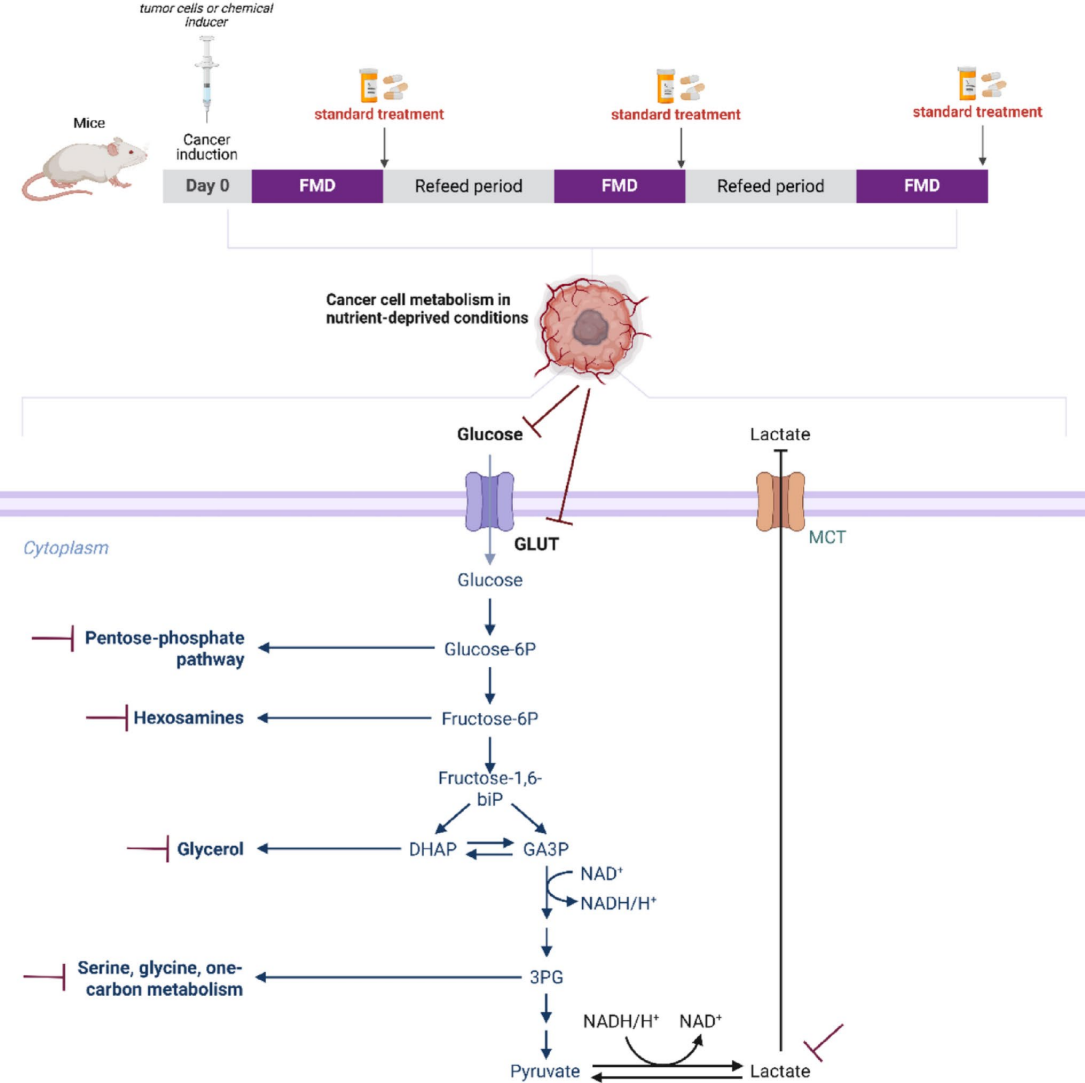
Antitumor action of FMD through glycolysis inhibition and warburg effect suppression

The fasting-mimicking diet (FMD) downregulates glycolysis, inhibits the Warburg effect, and promotes an antitumor response by shifting cellular metabolism toward oxidative pathways (Fig.8).In line with these metabolic effects, Table 6 summarizes the purpose and mechanisms of action of the therapeutic interventions used in association with FMD, illustrating how these strategies target complementary pathways related to energy metabolism, cell survival, oxidative stress, and immune modulation, thereby reinforcing the rationale for their combined use in preclinical cancer models [64-94].

Taken together, the findings of this review show that FMD acts simultaneously on metabolic, hormonal, immunological, and oxidative stress axes, broadly and multifacetedly reprogramming the tumor environment. The main limitation of this review is the heterogeneity among the included studies, which differ in animal models, protocol duration, number of cycles, therapeutic combinations, dosages, and evaluated outcomes. Nevertheless, in all preclinical scenarios, FMD demonstrated positive effects, reinforcing its translational potential in oncology. Future studies should focus on protocol standardization, defining the ideal duration, and identifying the most effective therapeutic combinations to maximize its clinical impact.

## Conclusion

This review highlights the therapeutic potential of fasting-mimicking diets in cancer management, providing a comprehensive synthesis of preclinical studies and the underlying mechanisms involved. FMD exhibits significant antitumor activity, particularly when used as an adjuvant to conventional therapies. It has been shown to reduce tumor volume and growth, enhance survival and immune response, downregulate circulating levels of IGF-1, insulin, and leptin, and increase the expression of pro-apoptotic markers such as caspase-3, while simultaneously reducing inflammatory mediators. These findings underscore FMDs role as a promising, non-pharmacological strategy capable of enhancing treatment efficacy, minimizing toxicity, and targeting tumor metabolism in a wide range of cancers.

## Supplementary Information

Below is the link to the electronic supplementary material.

Supplementary Material 1

## Data Availability

All data generated or analysed during this study are included in thispublished article.
