# Supplementary material for: Fasting-mimicking diets as a strategy to reprogram tumor metabolism: a systematic review
Source: Eur J Nutr. 2026 Feb 12;65(2):44. doi: 10.1007/s00394-026-03891-2 (PMC12901203; doi:10.1007/s00394-026-03891-2)
Supplement: Supplementary file 1 — Supplementary Material 1 [file 394_2026_3891_MOESM1_ESM.docx]

**Tabela Suplementar 1 –** Description of the purpose and mechanisms of action of the interventions used in association with the FMD

| Substance | Purpose of the medicine | Mechanism of action |
| --- | --- | --- |
| 2-deoxy-D-glucose (2-DG) | It is a non-metabolizable analogue of glucose in which the hydroxyl group in position 2 of glucose is replaced by hydrogen, with potential inhibition of glycolysis and antineoplastic activity [64]. | 2-DG inhibits the first step of glycolysis and prevents the cell from producing energy, resulting in a decrease in the proliferation of tumor cells [65]. |
| Anti- CTLA-4 | CTLA-4 is an inhibitory protein in activated T cells that competes with CD28 for the B7 ligands in antigen-presenting cells, inhibiting immune activation [66]. | Antibodies such as ipilimumab and tremelimumab block this interaction, allowing continued activation of T cells and strengthening the immune response against tumors. In addition, this therapy can reduce regulatory T cells (Tregs) in the tumor microenvironment, favoring a more effective antitumor response [66]. |
| anti-OX40 | It consists of an OX40 receptor agonist monoclonal antibody, with potential stimulatory activity [67] | Anti-OX40 activates the receptor and induces the proliferation of effector and memory T lymphocytes. In the presence of tumor-associated antigens, this may promote an immune response against TAA-expressing tumor cells [67]. |
| Abraxane | It is an albumin-bound paclitaxel. It is a chemotherapy drug used to treat various types of cancer, such as metastatic breast cancer, ovarian cancer and advanced non-small cell lung cancer [68]. | It works by inhibiting the depolymerization of microtubules, blocking cells in the G2 and M phases of the cell cycle, resulting in cell death. It is known as a mitosis inhibitor [69]. |
| Apatinib | It is a tyrosine kinase inhibitor (TKI) indicated for the treatment of various solid tumors, such as non-small cell lung cancer [70]. | Apatinib exhibits antigenic effects by inhibiting VEGFR-induced proliferation and migration of endothelial cells through highly selective targeting of VEGFR2 [71]. |
| Bortezomib | It is a proteasome inhibitor used in the treatment of cancers such as multiple myeloma [72]. | It blocks the 26S proteasome, preventing the degradation of essential proteins, which leads to the accumulation of defective proteins, causing cellular stress and inducing apoptosis. In addition, it inhibits the NF-κB pathway, reducing tumor cell survival [72]. |
| Cyclophosphamide | Is an alkylating agent belonging to the group of oxazaphosporines. Used in the treatment of lymphoma, leukemia, breast, ovarian, and small cell lung cancer [67]. | It exerts its effects through the alkylation of DNA. The drug is not cell cycle phase-specific and metabolizes to an active form capable of inhibiting protein synthesis through cross-linking of DNA and RNA [73] |
| Chloroquine | It is an aminoquinoline agent, commonly used to treat malaria and rheumatoid and which is being identified as a potential antitumor agent[74] | Chloroquine can penetrate the membrane and intercalate into double-stranded DNA without causing physical damage to the DNA and also accumulates in lysosomes and can induce the process of apoptosis by inhibiting the degradation of autophagic proteins [75] |
| Doxorrubicin | It is an antibiotic from the anthracycline family, extracted from *Streptomyces peucetius*, which has an antitumor effect against most cancers, such as breast, lung, leukemia, brain and lymphoma [76]. | It acts through intercalation into DNA and interruption of DNA repair mediated by topoisomerase II. The second mechanism involves the induction of oxidative stress to cell membranes, DNA and proteins [77]. |
| Fulvestran | It consists of a competitive estrogen receptor (ER) antagonist with an affinity comparable to that of estradiol [78]. | It works by binding, blocking and degrading ER, which in turn leads to the inhibition of estrogen signaling through estrogen receptors in the body. Fulvestrant blocks AF-1 and AF-2, blocking the transcriptional activities of both downstream and preventing estrogen from exerting its function [79]. |
| Ipatasertib | It is a highly selective oral ATP-competitive small molecule inhibitor of all three isoforms of AKT [80]. | It acts by binding and inhibiting the activity of Akt in a non-competitive manner with ATP, resulting in the inhibition of the PI3K/Akt signaling pathway and the proliferation of tumor cells and the induction of apoptosis of tumor cells [80]. |
| Metformin | It is a widely prescribed antihyperglycemic agent for the treatment of type 2 diabetes (T2D) [81]. | Acts as a suppressor of hepatic gluconeogenesis by inhibiting mitochondrial glycerophosphate dehydrogenase, altering the hepatic redox state to reduce the formation of glucose from lactate and glycerol [82] |
| Oxaliplatin | It is an alkylating agent and has non-cell cycle cytotoxicity. It is a platinum-based chemotherapy drug [83] | The platinum complex in the drug binds to DNA and forms cross-links. Cross-links inhibit DNA replication, transcription, and cell cycle arrest, resulting in cell death [83] |
| Palbociclib | It is a small molecule inhibitor of CDK4 and CDK6 [84]. | Inhibits the phosphorylation of retinoblastoma protein at the beginning of the G1 phase, leading to cell cycle arrest. This suppresses DNA replication and decreases tumor cell proliferation [84]. |
| Pictilisib | Is a small molecule inhibitor of class I phosphatidylinositol 3 kinase (PI3K), with potential antineoplastic activity [85]. | It selectively binds to PI3K in an ATP-competitive manner, inhibiting PIP3 production and activation of the PI3K/Akt signaling pathway. Inhibits the growth, motility and survival of tumor cells [85]. |
| Rapamycin | It is a macrolide produced by the bacteria Streptomyces hygroscopicus, which inhibits mTOR [86]. | It is a potent inhibitor of antigen-induced proliferation of T cells, B cells and antibody production [87] |
| Rituximabe | Rituximab is a chimeric monoclonal antibody (part human, part murine) that acts against the CD20 antigen, present on the surface of B lymphocytes. It is mainly used in the treatment of lymphomas, chronic lymphocytic leukemia [88]. | It binds specifically to CD20, a membrane protein expressed on mature B cells. Binding triggers the destruction of these cells by different pathways: antibody-dependent cell-mediated cytotoxicity; complement-dependent cytotoxicity and apoptosis [88]. |
| Tamoxifen | It is a selective estrogen receptor modulator [78] | Acts by blocking E2-mediated AF2 activity, resulting in ER antagonistic activity [78] |
| Vincristine | It consists of an indole dimer obtained from the extraction of periwinkle leaves from the chloranthaceae family, it is used in the treatment of acute lymphoblastic leukemia, breast cancer, colon cancer, lung cancer, soft tissue sarcomas [89]. | It acts by inhibiting the polymerization of tubulin and its incorporation into microtubules, preventing the assembly of the mitotic spindle and leading to an attenuation of mitosis and inducing apoptosis [90, 91] |
| Vitamin C | It is a water-soluble vitamin, also known as ascorbic acid, and is synthesized by all plants and most animals [92] | It acts as a reductant, that is, it donates an electron to a substrate while it itself is oxidized to an ascorbyl radical, a relatively stable free radical [93] |
| WZB117 | It is a glucose transporter 1 inhibitor (GLUT-1) [94] | It acts to inhibit glucose transport in cancer cells in a dose-dependent manner by negatively regulating glycolysis, induces cell cycle arrest and inhibits the growth of cancer cells [94]. |

**Legend**: 2-DG: 2-deoxy-D-glucose; T2D: Type 2 Diabetes; PI3K: Phosphatidylinositol 3-kinase; Akt: Protein kinase B; ATP: adenosine triphosphate; ER: Estrogen receptor; CP: cyclophosphamide; GLUT-1: glucose transporter type 1; WZB117: 2-Fluoro-6-(m-hydroxybenzoyloxy) Phenyl m-Hydroxybenzoate; OXO: Oxaliplatin; DNA: deoxyribonucleic acid; RNA: ribonucleic acid; TAA: Tumor-associated antigens; AF-1: activating function 1; AF-2: activating function
